# Association of Oral Tobacco-Free Nicotine Delivery Product with Acute Renal Tubular Necrosis

**DOI:** 10.3390/medicina60111846

**Published:** 2024-11-09

**Authors:** Ratna Acharya, William Clapp, Kiran Upadhyay

**Affiliations:** 1 Department of Pediatrics, Nemours Children’s Hospital, Orlando, FL 32827, USA; 2 Division of Anatomic Pathology, Department of Pathology, University of Florida, Gainesville, FL 32610, USA; 3 Division of Pediatric Nephrology, Department of Pediatrics, University of Florida, Gainesville, FL 32610, USA

**Keywords:** oral nicotine delivery product, acute tubular necrosis, kidney transplant, adolescents

## Abstract

Usage of novel non-tobacco oral nicotine delivery products (ONDPs) has been increasing among adolescents in the United States. It is presumed that they are less toxic than their tobacco-containing counterparts, but that has not been examined in controlled studies. Most of the studies have focused on non-renal manifestations of tobacco consumption via different means. The renal manifestations of non-tobacco ONDPs are not very well known, especially in immunocompromised patients. A 19-year-old male transplant recipient presented with flank pain and a few days’ history of intake of ZYN^R^ pouches. Immunosuppression was with tacrolimus, mycophenolate, and steroids. Baseline serum creatinine was 1.1–1.3 mg/dL. Laboratory evaluation showed elevated C-reactive protein, increased serum creatinine and blood urea nitrogen, leukocytosis, neutrophilia, and increased lactate dehydrogenase (LDH). Infectious disease work-up was negative. A kidney transplant biopsy showed severe acute tubular injury/necrosis (ATN) without evidence of rejection. Donor-specific antibodies were negative. Other etiologies of hemolysis were negative. He did not require renal replacement therapy. Kidney function and LDH improved gradually. The most recent follow-up eight months after presentation showed a serum creatinine level of 2.6 mg/dL with stable electrolytes, with eGFR of 35 mL/min/1.73 m^2^. Here, we describe a 19-year-old adolescent with a kidney transplant who sustained ATN leading to advanced chronic disease apparently following the usage of non-tobacco ONDP. Further larger studies are needed to study ATN as a possible renal manifestation of these next-generation products to raise awareness among the public.

## 1. Introduction

Oral nicotine delivery products (ONDPs) provide an alternate route of nicotine delivery to the conventional inhaled tobacco products [1]. In 2024, 1.8% of middle and high school students in the United States reported current usage of nicotine pouches [1]. Among those who currently used nicotine pouches, 29.3% reported frequent use, and 22.4% reported daily use. The majority of them used ZYN^R^ (68.7%); 85.6% used flavored products [2]. Some of the smokeless ONDPs currently available in the market are chewing tobacco, snus, and moist snuff. These smokeless ONDPs are known to contain several harmful and potentially harmful constituents (HPHCs) such as tobacco-specific nitrosamines and polycyclic aromatic hydrocarbons (PAHs) [3]. Various substances are used in these ONDPs, such as artificial sweeteners (xylitol, maltitol, sucralose, acesulfame K, aspartame), binders, humectants, pH adjusters, stabilizers, artificial colors, and coating agents [3]. Some of the additives, such as aspartame, have been linked with cancers such as hepatocellular carcinoma in humans [4].

Nicotine replacement therapies (NRTs), such as lozenges, gums, and patches, which predominantly deliver nicotine are widely used as treatments for tobacco addiction. Recently, tobacco-free nicotine pouches such as ZYN^R^ have also been gaining popularity among teens and adults as novel ONDPs. The ZYN^R^ pouch ingredients consist of water, fillers (microcrystalline cellulose and plant fibers), a humectant (glycerine), pH adjusters (sodium carbonate and calcium chloride), sodium chloride, food-grade flavorings, a nicotine solution, a monoglyceride, and a sweetener such as acesulfame K [5]. Also, a total of three HPHCs were found in both the ZYN^R^ dry and ZYN^R^ moist products: formaldehyde, chromium, and ammonia. Traces of nickel, just above the quantification limit, were also found, but nitrosamines and PAH were absent in ZYN^R^ dry [5]. Formaldehyde has been shown to cause glomerular degeneration, acute tubular necrosis, edematous obstruction, and hematuria [6].

Various studies have described nicotine-induced nephrotoxicity. Here, we present a 19-year-old male with a kidney transplant who presented with acute tubular necrosis after consumption of ZYN^R^. To the best of our knowledge, this association has not been described in the past.

## 2. Case Presentation

A 19-year-old Caucasian male underwent a living-related kidney transplant from his mother 17 years ago for end-stage kidney disease secondary to posterior urethral valve. He had stable allograft function throughout the years, with a baseline serum creatinine of 1.1–1.3 mg/dL (MDRD eGFR 71–96 mL/min/1.73 m^2^). He was last seen in the clinic for a follow-up about a month ago with stable baseline serum creatinine. He presented to the emergency department with sudden-onset right-sided flank pain associated with nausea and dysuria. He denied history of gross hematuria, decreased oral fluid intake, changes in urine output, trauma, vomiting, diarrhea, or fever. His medications consisted of tacrolimus 2 mg twice daily, mycophenolate 500 mg twice daily, prednisone 5 mg daily, and losartan 25 mg daily. He reported compliance with these medications. He admitted that he had started taking ZYN^R^ pouches (Swedish Match, Stockholm, Sweden) several days prior to this presentation, but denied smoking and usage of other tobacco-containing ONDPs. He also denied usage of non-steroidal anti-inflammatory drugs.

His past medical history was significant for hospitalization three months ago for acute left eyelid swelling in the setting of a history of chronic pansinusitis, nasal polyposis, and asthma for more than 10 years. The computed tomography scan of the orbit without contrast and magnetic resonance imaging of the sinuses with gadolinium showed evidence of extensive paranasal sinus disease with a dehiscence along the left cribriform plate with intracranial extension. He was treated with a three-day course of 60 mg daily prednisone followed by a taper to the baseline dose, ampicillin–sulbactam, levofloxacin, metronidazole, and fluconazole, for three weeks. Four weeks later, he underwent an extensive functional endoscopic sinus surgery along with bilateral ablation of nasal polyps. The tissue biopsy from both maxillary sinuses showed features of chronic rhinosinusitis with eosinophils and eosinophilic mucin, and Grocott methenamine silver (GMS) stains showed abundant fungal hyphae consistent with allergic fungal sinusitis. This was consistent with allergic fungal chronic rhinosinusitis rather than invasive fungal disease. There was no drainable sinus fluid to send for aspergillus or mucormycetes cultures. Chest x-ray was normal. The patient did not have clinical signs or symptoms of Pneumocystis jirovecii pneumonia (PJP), and hence, bronchoalveolar lavage was not felt to be necessary by the expert infectious disease team. Serum (1,3)-beta-D-glucan testing was negative.

Family history was not suggestive of kidney failure, dialysis, or transplantation, except for his mother being the donor for his transplanted kidney.

Upon presentation, his initial vital signs showed a temperature of 98.4 °F (36.8 °C), a heart rate of 110 beats per minute, a respiratory rate of 20 per min, a blood pressure of 104/66 mm Hg, an oxygen saturation of 95% on room air, and a body mass index of 24.1 kg/m^2^. Physical examination was otherwise normal except for an ill-appearing young male with a midline abdominal scar from prior kidney transplantation and mild tenderness in the right lower quadrant (RLQ).

Initial investigations showed elevated C-reactive protein (CRP), increased serum creatinine and blood urea nitrogen, leukocytosis, neutrophilia, and increased lactate dehydrogenase (LDH, Table 1). Plasma haptoglobin was normal. Human rhino/enterovirus was positive. Blood and urine cultures (obtained via clean catch) were negative. Renal transplant sonogram with doppler showed an echogenic transplant kidney measuring 10.9 cm in length with no hydronephrosis or perinephric fluid collection and patent waveforms in the renal artery and vein. Native kidneys were atrophic. A computed tomography scan of the abdomen without contrast showed a non-obstructing 3 mm stone in the mid pole of the RLQ transplant kidney. Urinalysis showed glycosuria without associated hyperglycemia, 2+ proteinuria, absence of microscopic hematuria, dilute urine, and no evidence of urinary tract infection. The random urine protein to creatinine ratio was 0.8. Urine beta-2 microglobulin was elevated (Table 1). All other infectious disease work-ups were negative.

Given suspected bacteremia due to the elevated CRP, the patient received a three-day course of intravenous cefepime, which was discontinued after the cultures resulted negative. He also received intravenous pulse methylprednisolone 1 gm daily for three days (prior to the biopsy) for suspected rejection.

**Figure 1:**
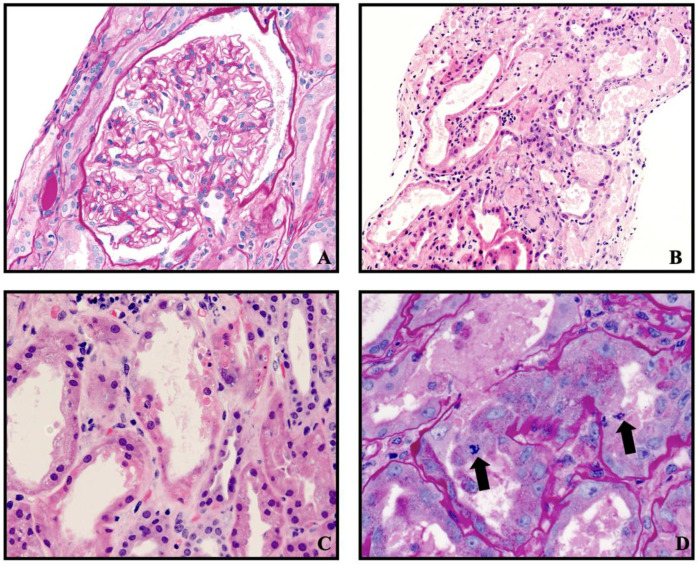
(**A**–**D**) showing the histologic images of the kidney transplant biopsy. (**A**) Normocellular glomerulus (PAS, 400×). (**B**) Acute tubular injury: tubular dilatation, cell sloughing, and casts (H&E, 200×). (**C**) Acute tubular injury: individual tubular cell necrosis (H&E, 400×). (**D**) Acute tubular injury: tubular epithelial mitotic figures (arrows) (PAS, 630×).

A percutaneous kidney transplant biopsy obtained three days after admission showed nine glomeruli which were normocellular with patent capillary lumens. There was no segmental sclerosis, necrosis, or crescent formation. Two of the glomeruli were globally sclerotic. There was interstitial fibrosis and tubular atrophy involving 25% of the cortical parenchyma. There were a few scattered CD3-positive lymphocytes and CD68 immunoreactive mononuclear cells, primarily within the interstitium (Figure 1). There was no peritubular capillaritis or tubulitis. There was severe acute tubular injury characterized by tubular cytoplasmic vacuolization; cell sloughing; individual cell necrosis; and large, regenerative-appearing nuclei, mitotic figures, and luminal casts (Figure 1A,D). There was prominent hyalinosis of the arterioles and a few small arteries. There were no definitive microthrombi or vasculitis identified. Immunostain for BK virus was negative. Immunofluorescence microscopy showed that glomeruli were negative for IgG, IgM, IgA, C3, C1q, albumin, kappa light chain, and lambda light chain. Electron microscopy showed a renal cortex exhibiting acute tubular and glomerular endothelial cytoplasmic swelling along with mild mesangial expansion, without any electron-dense deposits. The donor-specific antibodies were negative.

**Figure 2:**
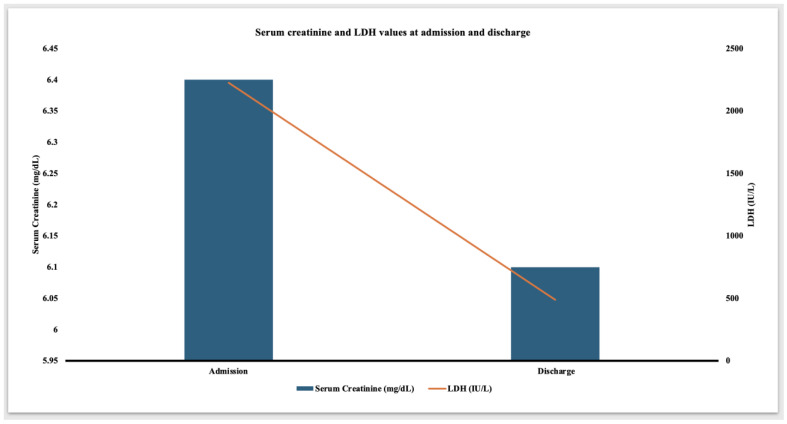
Graph showing the serum creatinine and serum LDH values upon admission and at discharge.

Given the lower likelihood of rejection, the steroid dose was converted to a home baseline dose of 5 mg daily, and he did not receive further anti-rejection therapies. Losartan was switched to amlodipine due to elevated serum creatinine. The patient remained afebrile and hemodynamically stable. CRP peaked at 389 mg/L on day four of presentation. Serum LDH showed a gradual decline. Serum trough tacrolimus levels remained therapeutic, with a goal trough level of 3–5 ng/mL. The abdominal pain and hematuria resolved about a week after presentation. At the time of discharge, serum LDH decreased to 488 IU/L (Figure 2) and normalized three weeks later. Serum creatinine was 6.1 mg/dL at the time of discharge and was followed up closely on an outpatient basis. The discharge medications consisted of tacrolimus 2 mg twice daily, mycophenolate 250 mg twice daily, prednisone 5 mg daily, and amlodipine 10 mg daily.

A repeat transplant kidney biopsy four months after initial presentation showed evidence of severe interstitial fibrosis involving about 80% of the renal cortex, evidence of grade 1A T cell mediated rejection, negative BK virus staining, and severe interstitial fibrosis/tubular atrophy involving 80% of the parenchyma. Given the age of the transplant and severe chronicity, the clinical team and the family decided not to augment the immunosuppressive therapy. The most recent follow-up eight months after presentation showed a serum creatinine level of 2.6 mg/dL with stable electrolytes, with eGFR of 35 mL/min/1.73 m^2^.

## 3. Discussion

ZYN^R^ is a novel, next-generation, non-tobacco-based ONDP in a pouch formulation which does not burn tobacco or produce smoke. Studies have shown that there are no measurable levels of nitrosamines or PAHs in these products and that the numbers of quantified HPHCs are similar between ZYN^R^ and NRT products, found at low levels compared to tobacco-based products [5]. The incidence of usage of nicotine in high schoolers and young adults remains substantial. In 2023, 22.2% of U.S. middle and high school students reported ever using any tobacco product, and 10% of students reported current use of any tobacco product [7]. With regard to the nicotine pouches, their current usage was 41.3% among the current smokeless tobacco users [8].

Nicotine has been shown to be nephrotoxic [9,10,11]. The kidneys of chronic smokers contain high concentrations of nicotine via glomerular filtration and tubular secretion of nicotine [10,12]. The human proximal renal tubular epithelial cells (PRTECs) have prominent nicotinic acetylcholine receptors (nAChR) [13,14]. One study showed that nicotine causes nephrotoxicity through the induction of NLRP6 inflammasome and alpha7 nAChRs [13]. The mean renal tubular injury scores were higher in the nicotine group than in the normal group [13]. In this study, the expression of kidney injury molecule-1 (KIM-1), a marker of tubular injury, was also significantly increased in the kidney tissue [13]. Nicotine also has been shown to induce apoptosis by generating reactive oxygen species and cell cycle arrest at the G2/M phase, and by activating the MAPK and NF-κB signaling pathways in PRTECs [15]. The PRTECs are highly susceptible to apoptosis, leading to acute tubular injury [16]. The tubular injury seen in the patient described in this report could have been from one of these mechanisms. Repeated nicotine exposure from chronic smoking may sensitize the kidney to ischemic insults and may facilitate the progression of acute kidney injury to chronic kidney injury [17].

LDH is commonly measured in patients suspected to have hemolysis. However, it is also a marker of cell injury and necrosis. A high concentration of LDH is found in the renal tubular cells [18]. Hence, the elevated level of serum LDH may be a biomarker of renal tubular injury [19,20,21]. After kidney transplantation, ischemic ATN, not allograft rejection, leads to a rise in LDH [22,23]. In delayed graft function, LDH may act as a good non-invasive marker of kidney injury and may serve as a better way to serially assess the clinical outcome or guide the management [24,25]. In kidney transplant recipients with preexistent tubular atrophy and interstitial fibrosis, such as this patient, ONDPSs may exacerbate the renal tubular injury, leading to marked elevation in LDH. However, this needs to be studied further. Tacrolimus is a known cause of drug-induced hemolytic uremic syndrome (HUS). However, in the absence of peripheral schistocytes, thrombocytopenia, decreased plasma haptoglobin, absence of thrombi in the biopsy, and normal serum trough tacrolimus levels, tacrolimus-induced HUS leading to elevated LDH was unlikely 17 years after the transplantation. In the absence of anemia, normal plasma haptoglobin, and a normal Coombs test, other causes of transient elevation in serum LDH were less likely in the patient described in this report. The serum LDH and (1,3)-beta-D-glucan are good non-invasive markers of PJP infection in HIV-uninfected immunocompromised patients [26]. However, given the absence of clinical features of PJP and normal (1,3)-beta-D-glucan, this was unlikely in this patient. One of the limitations of the study is the unavailability of urinary cotinine levels, along with the immunohistochemical markers of the nicotine and ZYN^R^ metabolites in the kidney tissue specimen.

## 4. Conclusions

This report highlights the importance of knowledge of the possible association of ONDPs with acute tubular injury. It remains to be studied whether the incidence of this specific renal toxicity is more prevalent in immunocompromised patients, as described in this report. Although we could not establish a definite cause and effect relationship between ONDP and renal tubular injury, this report suggests that clinicians will need to be vigilant in searching for a history of usage of ONDPs in patients presenting with unexplained rises in LDH and acute tubular injury. Given the widespread usage among high schoolers, further studies are necessary to determine the incidence of acute tubular injury in users of ONDPs.

## Figures and Tables

**Table 1:** Laboratory values at presentation and discharge. ANA: antinuclear antibody; ANCA: anti-neutrophil cytoplasmic antibody; BUN: blood urea nitrogen; C3: complement 3; C4: complement 4; CMV: cytomegalovirus; CK: creatine kinase; CRP: C-reactive protein; EBV: Epstein–Barr virus; LDH: lactate dehydrogenase.

	Initial	Discharge at 3 Weeks
BUN	46 mg/dL	96 mg/dL
Serum creatinine	6.4 mg/dL	6.1 mg/dL
Serum albumin	3.5 g/dL	3.4 g/dL
CRP	384.5 mg/L (0–5 mg/L)	35.5 mg/L
LDH	2227 IU/L (Normal: 135–225 IU/L)	488 IU/L
Haptoglobin	156 mg/dL (Normal: 40–215 mg/dL)	
Peripheral smear schistocytes	Absent	
Peripheral blood flowcytometry	Negative for malignancy	
Direct Coombs test	Negative	
C3	134 mg/dL	
C4	13 mg/dL	
ANA	Negative	
ANCA	Negative	
CK	<10 U/L	
WBC	22.9 × 10^3^/uL with left shift, neutrophils 85.6%	
Hemoglobin	13.2 g/dL	
Platelet count	175 × 10^3^/uL	
BK DNA PCR	Not detected	
CMV DNA PCR	Not detected	
EBV DNA PCR	Not detected	
Urinalysis	pH 7, specific gravity 1.010, red cells 4 per hpf, 2+ protein, positive glucose, negative nitrites and leukocytes	
Urine protein to creatinine ratio	0.8 mg/mg creatinine	
Urine beta-2 microglobulin	30,000 mcg/L (≤300 mcg/L)	
Urine toxicology	Negative	

## Data Availability

The authors declare that data supporting the findings of this study are available within the article.
